# Cytomegalovirus Infection After Solid Organ Transplantation: How I Use Cell-Mediated Immune Assays for Management

**DOI:** 10.3390/v16111781

**Published:** 2024-11-15

**Authors:** Raymund R. Razonable

**Affiliations:** Division of Infectious Diseases and the William J von Liebig Center for Transplantation and Clinical Regeneration, Mayo Clinic, Rochester, MN 55905, USA; razonable.raymund@mayo.edu; Tel.: +1-507-2557761

**Keywords:** cytomegalovirus, cell-mediated immunity, interferon gamma, transplantation, solid organ transplantation, immunosuppression, antiviral therapy, relapse, prophylaxis, outcomes

## Abstract

Introduction: The pathogenesis and outcome of cytomegalovirus (CMV) infection after solid organ transplantation (SOT) reflects the interplay between viral replication and CMV-specific immunity. Despite advances in its diagnosis and treatment, CMV continues to cause significant morbidity after SOT. Since CMV is an opportunistic pathogen that occurs as a result of impaired pathogen-specific immunity, laboratory assays that measure CMV-specific immune responses may be useful in assisting clinicians in its management. Methods and Results: The author summarizes the evolving and emerging data on the clinical utility of assays that quantify cell-mediated immune responses to CMV in SOT recipients. The majority of publications are observational studies that demonstrate that a lack or deficiency in CMV-specific cell-mediated immunity is correlated with a heightened risk of primary, reactivation, or recurrent CMV after transplantation. A few prospective interventional studies have utilized CMV-specific cell-mediated immune assays in guiding the duration of antiviral prophylaxis among CMV-seropositive SOT recipients. Likewise, CMV-specific cell-mediated immunity assays have been suggested to inform the need for secondary antiviral prophylaxis and immunologic optimization to prevent CMV relapse after treatment. Conclusions: CMV-specific cell-mediated immune assays are emerging to assist transplant clinicians in predicting a patient’s risk of CMV after transplantation, and these assays have been utilized to individualize the approach to CMV prevention and treatment. The author suggests the conduct of more interventional studies to further solidify the role of CMV-specific cell-mediated immune assays in routine clinical practice.

## 1. Introduction

Cytomegalovirus (CMV) is a ubiquitous herpesvirus that infects the majority of adults [1]. When an immunocompetent person acquires CMV infection, it is generally a self-limiting, non-specific illness that spontaneously resolves without antiviral therapy. CMV-specific cellular and humoral immunity develops during primary CMV infection, characterized by the generation of CMV-specific CD4+ and CD8+ T lymphocytes and CMV-IgM and IgG antibodies, respectively [2]. However, CMV persists lifelong in humans, with periodic reactivations, but the virus is kept in a latent subclinical state by a functioning cell-mediated immune system [3].

In persons with compromised immune systems, primary or reactivation CMV infection can lead to high morbidity and, if untreated, potential mortality. In the field of solid organ transplantation (SOT), where patients are given lifelong pharmacologic immunosuppression to prevent allograft rejection and ensure the survival of the transplanted allograft, CMV has the potential to cause severe disease [4]. The major target of most immunosuppressive drugs used in SOT recipients is the T lymphocyte, although other immune cells, such as natural killer (NK) cells, may also be affected. Some immunosuppressive drugs deplete T lymphocyte populations (lymphocyte-depleting drugs; e.g., thymoglobulin, alemtuzumab), while others impair lymphocyte function (e.g., basiliximab) [5]. SOT recipients are maintained lifelong on a combination of pharmacologic immunosuppressive drugs that impair the function of T lymphocytes and other immune cells, such as calcineurin inhibitors (tacrolimus, cyclosporine), antimetabolites (mycophenolate, azathioprine), and corticosteroids [4]. While these drugs are intended to ensure allograft survival, one major adverse outcome is a heightened risk of opportunistic infections, including CMV.

CMV infection is a common complication after SOT, and it can either be primary infection or reactivation of latent virus [4]. Primary CMV infection can be potentially severe and life-threatening in SOT recipients. Most often, primary CMV infection occurs when CMV-seronegative transplant patient receives an organ from a CMV-seropositive donor (CMV D+/R− mismatch) [4]. Among CMV-seropositive SOT recipients with pre-existing immunity, latent CMV may reactivate to cause clinical disease when there is depletion or marked impairment in the function of T lymphocyte populations [4]. CMV disease may be manifested as fever associated with malaise, leukopenia, thrombocytopenia, and mild hepatic transaminitis (CMV syndrome) [4]. In many patients, CMV may be disseminated to various organs to cause dysfunction, including the transplanted allograft. The gastrointestinal tract is the most commonly affected organ system [6], while CMV pneumonitis can be particularly severe, morbid, and potentially fatal [7]. CMV may affect any organ system, including the central nervous system (CNS) and the retina (i.e., retinitis) [8]. CMV has been correlated with poor allograft and patient survival after SOT [9].

Because of the negative effects of CMV in SOT, its prevention is part of standard of care. This can be accomplished either through antiviral prophylaxis or preemptive therapy [4]. Antiviral prophylaxis entails the administration of an antiviral drug, most commonly valganciclovir or letermovir, for a defined period (usually as short as 3 months to as long as 12 months, or longer) after SOT [4]. In contrast, preemptive therapy is a strategy that entails antiviral drug administration only upon the detection of asymptomatic CMV replication. Using this strategy, patients are monitored regularly, often once weekly, via CMV nucleic acid amplification testing (e.g., CMV DNA polymerase chain reaction [PCR]) of blood samples during the first 3 months after SOT. If CMV DNA is detected above a pre-specified viral load threshold, antiviral drugs such as valganciclovir are provided to treat the patients until the virus is no longer detectable in the blood [4].

The risk, clinical course, and outcome of CMV in SOT is dependent on the interplay between virus replication and host immune fitness. Virus replication is measured via quantitative CMV nucleic acid amplification testing, most commonly via CMV DNA PCR. For decades, the only clinical test of CMV-specific immunity has been serology, which has served as a backbone for pre-transplant immunologic screening of organ donors and transplant candidates. Recently, measures of CMV-specific cell-mediated immunity (CMV-CMI) have emerged from research laboratories to clinical settings. In this article, the author reviews the data on CMV-CMI and provides perspectives on the roles of these assays in the management of CMV in SOT recipients.

## 2. CMV-CMI Assays: Brief Overview

CMV-CMI assays are laboratory tests that measure activated T lymphocytes after ex vivo stimulation with CMV antigens. Most commonly, activated T lymphocytes are indicated by their ability to express or secrete cytokines, most commonly interferon gamma, after stimulation ex vivo with CMV antigens, most commonly pp65 and IE-1 antigens [10]. There are several CMV-CMI assay platforms that are available from commercial and research laboratories, including an interferon gamma release assay (IGRA; QuantiFERON-CMV [Qiagen, Inc., Hilden, Germany]) [11,12,13,14,15] and quantitation of interferon gamma-expressing cells per pre-determined number of peripheral blood mononuclear cells (PBMC), either through enzyme-linked immunosorbent spot assays (ELISPOT; e.g., T-SPOT.CMV [Oxford Diagnostics, Abingdon, Oxfordshire, United Kingdom] [12], T-Track-CMV [Lophius Biosciences, Regensburg, Germany]) [16,17] or intracellular cytokine staining through flow cytometry (e.g., CMV inSIGHT T cell immunity testing [Viracor Eurofins, Lenexa, Kansas] [18,19], CMV CD8+ T cell immune competence assay [Mayo Clinic Laboratories, Rochester, MInnesota] [20]). There are other laboratory-developed tests (LDT) that utilize these similar principles of measuring T lymphocyte activation ex vivo in response to CMV antigens [21].

CMV-CMI can measure CD4+ and/or CD8+ T lymphocytes using whole blood or isolated PBMC samples. After collection, blood samples are incubated for a defined period of time (e.g., overnight incubation) in the presence of CMV-specific peptides [19]. Thereafter, the amount of secreted interferon gamma in plasma (QuantiFERON-CMV) [5,11,13,15] or the number of interferon-producing cells (T-SPOT.CMV, T-Track-CMV, flow cytometry) are measured [12,16,19,20]. The results of the CMV-CMI assays are interpreted in the presence of a negative and a positive (mitogen) control; these are included to ensure quality of CMV-CMI testing.

However, there is lack of standardization across the different CMV-CMI assays. The variability in performance among the CMV-CMI assays may be related to differences in their methods, antigenic stimulants, clinical samples, and reporting parameters, among others. Furthermore, clinically available CMV-CMI assays do not fully reflect the full complement of CMV immunity. Most assays measure mainly activated CD8+ T lymphocytes, with interferon gamma production only as the read-out. Accordingly, other components of the CMV immune response, such as NK cells, are not accounted for. In vivo, all immune components are anticipated to synergistically act to control CMV; hence, the functional assessment of only one component (i.e., CD8+) does not fully reflect the global state of CMV-specific immunologic function.

## 3. Clinical Applications of CMV-CMI Assays

Detection of high levels of interferon gamma in plasma or high numbers of interferon gamma-producing cells (via ELISpot or flow cytometry) generally correlates with CMV-specific immunity and phenotypically, confering protection from CMV infection. Conversely, low levels or absence of these immune measures have been consistently associated with higher risk of CMV disease after SOT. With this general principle, Table 1 lists the potential clinical applications of CMV-CMI assays in the field of SOT, from the pre-transplant to the post-transplant period. The vast majority of clinical studies on CMV-CMI in SOT support its role as a prognosticator for CMV risk. Recently, there have been efforts to utilize CMV-CMI assays to guide decisions on antiviral prophylaxis, preemptive therapy, and treatment of disease. In a series of five questions, the author reviews the evidence supporting the use of CMV-CMI in the clinical setting.

### 3.1. Can I Use Pre-Transplant CMV-CMI to Predict the Risk of CMV After Transplantation?

CMV-IgG serology is the standard pre-transplant test used to determine a transplant candidate’s (and donor’s) prior CMV exposure and inform the risk after transplant. A negative CMV-IgG serology in a transplant recipient portends a very high risk of CMV disease after transplant if they receive an organ from a CMV IgG-seropositive donor (CMV D+/R− mismatch). In contrast, positive CMV-IgG serology in a transplant candidate correlates with pre-existing CMV immunity and portends a relatively lower risk (but not absolute protection) of CMV infection in the post-transplant period.

Recently, there have been emerging data illustrating that CMV-IgG does not always correlate with the presence of an effective CMV-CMI. In one study of 100 CMV-IgG-positive individuals, there was 18% disagreement between serology and CMV-CMI [22]. In another study of 44 CMV-seropositive lung and kidney transplant candidates, only 30 (68%) had detectable CMV-CMI, as measured by interferon gamma production [23]. In a large study of 583 kidney transplant recipients, about 8% of CMV-seropositive patients had undetectable or low-level CMV-CMI (T-SPOT.CMV) during the pre-transplant period [12].

Moreover, recent studies have demonstrated that absence of CMV-CMI in CMV-seropositive transplant candidates and recipients is significantly correlated with a higher risk of post-transplant CMV infection. In the study of 44 CMV-seropositive lung and kidney recipients, the rate of post-transplant CMV was significantly higher among patients with non-reactive versus reactive pre-transplant CMV-CMI (7 of 14 [50%] vs. 4 of 30 [13.3%], respectively; *p* = 0.021) [23]. Another study of 30 living donor liver transplant recipients demonstrated that 13 patients with positive pre-transplant CMV-CMI (QuantiFERON-CMV) had lower risk of CMV disease (15.4% vs. 58.8%; *p* = 0.016), faster clearance of viremia (7 days vs. 21 days; *p* = 0.004), and shorter duration of antiviral drug treatment (13 days vs. 28 days; *p* = 0.003) when compared to CMV-seropositive liver recipients with negative pre-transplant CMV-CMI [24]. In a prospective interventional clinical trial of 160 CMV-seropositive patients, those with undetectable or low pre-transplant CMV-CMI had significantly higher rates of post-transplant CMV infection [25]. Based on these observations, CMV-CMI may be considered as a supplement in the pre-transplant assessment of CMV-seropositive SOT candidates with the goal of stratifying patients into high or low risk of CMV after transplantation, potentially guiding CMV prevention strategies.

- *CMV-CMI may be considered as a supplement to CMV serology in the pre-transplant assessment of CMV-seropositive SOT candidates to better inform the risk of post-transplant CMV infection*.

In contrast, pre-transplant CMV-CMI may not be indicated in CMV-seronegative SOT candidates, since there is high concordance between CMV IgG-seronegativity and lack of CMV-CMI. In a prospective observational study that included 260 CMV D+/R− patients, only about 5% of CMV-seronegative individuals had any detectable CMV-CMI (T-SPOT.CMV) in pre-transplant specimens [12]. Since almost all CMV-seronegative persons are CMV-CMI non-reactive [11,12], routine assessment of pre-transplant CMV-CMI among CMV-seronegative transplant candidates is neither a practical nor cost-effective strategy.

- *CMV-CMI should not be routinely assessed during the pre-transplant screening of CMV-seronegative transplant candidates*.

### 3.2. When Can I Measure CMV-CMI After Transplantation to Predict the Risk of CMV Infection After SOT?

Pharmacologic immunosuppression after SOT may entail the use of T lymphocyte-depleting agents (e.g., anti-thymocyte globulin, alemtuzumab) and impair T lymphocyte function (e.g., basiliximab, mycophenolate, tacrolimus, steroids). The overall effect of these drugs is highest during the first 1–3 months after SOT. Accordingly, CMV-CMI measurement during this time period may be useful to inform the risk of CMV in the post-transplant setting, particularly among CMV-seropositive SOT recipients. However, the specific time points for CMV-CMI measurements after transplantation are neither well defined nor standardized.

Some studies have investigated CMV-CMI as early as 2 weeks after SOT, while others perform CMV-CMI testing serially, on a monthly basis, and others at the anticipated end of antiviral prophylaxis. Among CMV-seropositive patients, there is an expected profound abrogation of CMV-CMI during the first 2–4 weeks after SOT. This marked impairment in CMV-CMI is anticipated to occur in the majority of SOT patients, including those who did not receive T cell-depleting induction therapy, especially if the impairment in T cell function is severe. In one study that measured CMV-CMI (T-SPOT.CMV) on day 15 after transplantation [25], there was profound abrogation in total T lymphocyte counts, which was later found to be a predictor of subsequent CMV infection among patients treated with anti-thymocyte globulins [25]. Among the 124 CMV-seropositive kidney transplant recipients, low levels of CMV-specific CD8+ T lymphocytes (<2.0 cells/μL, measured via intracellular cytokine staining) on post-transplant day 15 had greater subsequent risk of CMV events [26]. Notably, another study reported that no patient with preserved (i.e., positive) CMV-CMI subsequently developed CMV disease [27]. Moreover, almost all patients with CMV-CMI controlled CMV reactivation without needing antiviral treatment [27].

Starting at 30 days after SOT, CMV-seropositive patients display progressive, albeit heterogenous, patterns of CMV-specific immune reconstitution [28]. In a study that included 70 CMV-seropositive SOT recipients, there was a steady and constant CMV immune reconstitution starting on day 60 [28]. In a large study that included 277 CMV-seropositive kidney transplant recipients, CMV-CMI (T-SPOT.CMV) was recovered in the majority of patients by 3 months after transplantation [12]. In another study of 78 CMV R+ and CMV-CMI-positive patients, 59.5% of the patients had recovered CMV-CMI by day 30, and 82.7% had recovered by day 90 [29]. Having lower counts of CMV-specific CD4+ T cells at days 60 and 180 were associated with a higher incidence of late-onset CMV events [26].

- *CMV-CMI measurement during the immediate post-transplant period may inform the risk of subsequent CMV infection among CMV-seropositive SOT recipients. In general, absence of CMV-CMI in CMV-seropositive SOT recipients is a risk factor for subsequent CMV infection. However, the frequency and time points of CMV-CMI measurements are not well defined*.

CMV D+/R− SOT recipients are at highest risk of CMV disease and are generally provided with antiviral prophylaxis for prolonged durations [4]. Failure to develop CMV-CMI (QuantiFERON-CMV) at the end of antiviral prophylaxis correlated with subsequent risk of CMV disease after completion of antiviral prophylaxis. In a cohort of 124 CMV D+/R− SOT recipients, the rate of subsequent CMV disease was 6.4% among those with reactive CMV-CMI, compared to 22.2% among those with non-reactive CMV-CMI [13].

Multiple studies have consistently demonstrated that CMV-CMI does not develop in the vast majority of CMV D+/R− SOT recipients during the period of antiviral prophylaxis. In one small study that included 13 CMV D+/R− SOT recipients, CMV-CMI (ELISPOT) was not attained during antiviral prophylaxis [28]. CMV-CMI (T-SPOT.CMV) at 3 months, when antiviral prophylaxis was discontinued, was undetectable in all 21 CMV D+/R− kidney transplant recipients [30]. Among the 49 CMV D+/R− kidney or pancreas transplant recipients who had CMV-CMI tested during antiviral prophylaxis, the majority (n = 46; 94%) had undetectable CMI (inSIGHT TCIP) [18]. In a cohort of 24 CMV D+/R− SOT recipients who received 6 months of valganciclovir, only 1 patient (4.2%) demonstrated CMV-CMI (QuantiFERON-CMV) after completion of valganciclovir prophylaxis [11]. In a study that evaluated CMV-CMI (T-SPOT.CMV) in a large cohort that included 257 CMV D+/R− kidney transplant recipients, very few developed CMV-CMI by the end of either 3 months or 6 months of antiviral prophylaxis [12]. Indeed, CMV-CMI was detected only after they have developed preceding post-prophylaxis CMV viremia [31]. Accordingly, CMV-CMI measurement is not routinely recommended during antiviral prophylaxis in CMV D+/R− SOT recipients.

- *CMV-CMI remains non-reactive (or negative) in the vast majority of CMV D+/R− SOT recipients during the period of antiviral prophylaxis. Thus, CMV-CMI measurement is not recommended among CMV D+/R− SOT recipients during the period of, and at the end of, antiviral prophylaxis*.

### 3.3. Can I Use CMV-CMI Measurement to Guide the Duration of Antiviral Prophylaxis?

Antiviral prophylaxis entails the administration of an antiviral drug for a defined period of time after SOT. It has been proposed, however, that the duration of antiviral prophylaxis may be individualized based on risk profile, as indicated by CMV-CMI assay. Ideally, the antiviral drug should be given until CMV immune reconstitution (i.e., reactive CMV-CMI) has occurred.

However, because CMV D+/R− SOT recipients do not generate CMV-CMI during the period of antiviral (valganciclovir) prophylaxis, CMV-CMI may not be a useful test to guide the duration of prophylaxis in these highest-risk patients [32]. In these patients, CMV-specific CD4+ and CD8+ T lymphocyte interferon gamma and polyfunctional responses were only generated after CMV replication has occurred. This observation illustrates the need for viral replication and antigen presentation in order to develop CMV-CMI, and this is not possible during complete viral suppression via valganciclovir prophylaxis. Accordingly, routine serial CMV-CMI measurements are not useful to guide the duration of antiviral prophylaxis among high-risk CMV D+/R− SOT recipients [33].

- *CMV-CMI measurement is not useful to guide the duration of antiviral prophylaxis among CMV D+/R− SOT recipients*.

In contrast, among CMV-seropositive SOT recipients, the profound abrogation of CMV-CMI, especially among those who received induction with T cell-depleting anti-thymocyte globulin, is only transient, and it is no longer evident in most patients 3 months after transplantation [34]. Most studies demonstrate that CMV reconstitution is achieved in the majority of CMV-seropositive SOT recipients by end of prophylaxis at 3 or 6 months (some even earlier), and this was protective from CMV disease. Multivariate binary logistic regression analysis revealed that lack of CMV-CMI at the time of prophylaxis cessation was the only independent correlate predicting CMV infection among CMV-seropositive SOT recipients [34]. In another study of 60 CMV-seropositive lung transplant recipients, poor CMV-CMI (T-SPOT.CMV) at 6 months (when antiviral prophylaxis was discontinued) was significantly correlated with subsequent post-prophylaxis CMV infection [35]. However, even as early as 30 days following SOT, some CMV-seropositive patients already displayed progressive, albeit heterogenous, patterns of CMV-specific immune reconstitution [28]. This raises the question: can CMV-CMI guide the duration of prophylaxis in CMV-seropositive SOT recipients?

In one open-label non-inferiority trial, 150 CMV-seropositive kidney recipients who received anti-thymocyte globulin induction were randomized to receive a standard, fixed duration of antiviral prophylaxis (up to 3 months) or to have the antiviral prophylaxis discontinued earlier upon the detection of CMV-CMI (QuantiFERON-CMV) [36]. With this strategy, CMV-CMI guidance led to earlier discontinuation of antiviral prophylaxis (median, 57 days) in the majority (59.2%) of patients. Despite shorter durations of antiviral prophylaxis, none of the 76 patients in the immuno-guided prophylaxis group developed CMV disease, compared to 2 of 74 patients who received standard 90-day prophylaxis. Because of the shorter duration of prophylaxis, the rate of neutropenia was significantly lower in the immune-guided prophylaxis group (9.2%, compared to 37.8% among those who received a fixed 90-day duration of prophylaxis) [36].

CMV-CMI (QuantiFERON-CMV) was also used to guide the length of antiviral prophylaxis after lung transplantation [14]. In this interventional study, 118 lung transplant recipients at risk of CMV infection (88 were CMV-seropositive and 30 were CMV D+/R−) were randomized to receive either 5 months of a fixed duration of prophylaxis or to have the duration individualized and extended up to 11 months depending on serial CMV-CMI measurements. The incidence of CMV infection in the lung allograft within 18 months after lung transplantation was significantly lower in the CMV-CMI-directed cohort (37% versus 58%, *p* = 0.03). Of the 80 patients who discontinued antiviral prophylaxis after a fixed duration of 5 months, the incidence of CMV DNAemia (>600 copies/mL) was significantly lower in the patients with a reactive versus non-reactive CMV-CMI (13% versus 67%, *p* = 0.0003). Likewise, the incidence of high-degree viral replication (>10,000 copies/mL) was significantly lower in patients with a reactive versus non-reactive CMV-CMI (3% versus 50%, *p* < 0.001). A non-reactive CMV-CMI after 11 months of antiviral prophylaxis was associated with a 25% incidence of CMV DNAemia [14]. In a retrospective study of 263 lung transplant patients, the majority of the 204 CMV-seropositive lung recipients (76%) achieved a reactive CMV-CMI after 5 months and had antiviral prophylaxis discontinued. CMV DNAemia was uncommon among those with reactive CMV-CMI [37].

**Figure 1:**
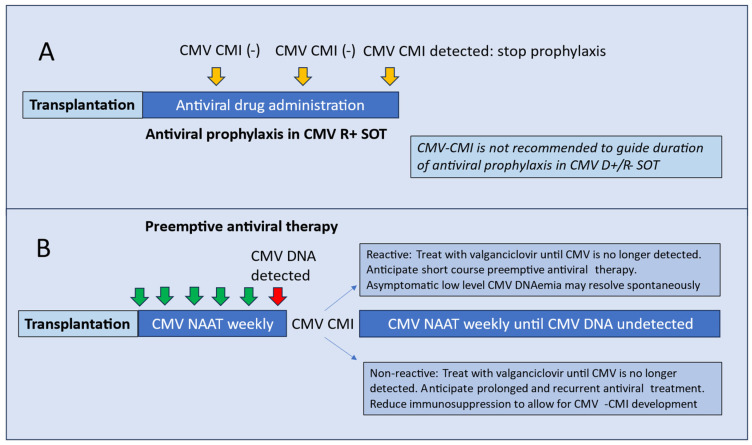
Strategies for cytomegalovirus (CMV) disease prevention and treatment that incorporates CMV-specific cell-mediated immunity. Top panel (**A**)—Antiviral prophylaxis: CMV-specific cell-mediated immunity may be measured during the period and at the anticipated end of prophylaxis among CMV-seropositive solid organ transplant recipients; antiviral prophylaxis may be safely discontinued when CMV-specific cell-mediated immunity is detected in CMV-seropositive solid organ transplant recipients. This approach is not applicable to CMV D+/R− solid organ transplant recipients, since CMV-specific cell-mediated immunity is rarely attained during antiviral prophylaxis. Bottom panel (**B**)—CMV surveillance and preemptive therapy: CMV-specific cell-mediated immunity may be measured at the time of CMV reactivation. CMV-immune patients with low-level viral reactivation may resolve the infection spontaneously or require a short course of preemptive antiviral treatment. CMV non-immune persons are anticipated to need a longer course of antiviral treatment. Footnote: CMV, cytomegalovirus; CMI, cell-mediated immunity; NAAT, nucleic acid amplification test.

These observations collectively suggest that reconstitution of CMV-CMI occurs in the majority of CMV-seropositive patients during the course of standard antiviral prophylaxis, and many even prior to the anticipated end date of antiviral prophylaxis. Accordingly, one may consider performing serial CMV-CMI to determine the time to CMV immune reconstitution, when antiviral prophylaxis may be discontinued (Figure 1A).

- *Serial CMV-CMI measurements may be performed during antiviral prophylaxis among CMV-seropositive SOT recipients. Detection of reactive CMV-CMI suggests immune reconstitution that allows for earlier discontinuation of antiviral prophylaxis*.

Conversely, the absence of CMV-CMI at the anticipated end of standard-duration antiviral prophylaxis (3–6 months) confers a higher risk of CMV infection and warrants the institution of efforts to prevent CMV reactivation. This may be in the form of extended or prolonged prophylaxis, aggressive CMV surveillance post-prophylaxis, and importantly, consideration to minimize the degree of pharmacologic immunosuppression, if feasible, to allow for CMV-CMI reconstitution [34].

### 3.4. Can I Use CMV-CMI in Guiding the Decision to Treat SOT Recipients with Asymptomatic CMV Reactivation?

Prevention of CMV disease after SOT can be accomplished by preemptively treating asymptomatic CMV infection with oral valganciclovir or intravenous ganciclovir; this is known as a preemptive therapy approach. However, the viral load threshold that triggers antiviral therapy in asymptomatic SOT patients is not known.

Observational studies have reported that asymptomatic CMV reactivation in transplant patients with a robust CMV-CMI are often low-level, transient, and may resolve spontaneously. In one study, CMV-CMI (QuantiFERON-CMV) was measured in 37 SOT recipients with asymptomatic CMV viremia (mean viral load, 1140 copies/mL). Among the 26 patients (70.3%) with a reactive CMV-CMI at the onset of viremia, 24 had spontaneous clearance of viremia without antiviral therapy [38]. Of interest, in a case series of 12 CMV D+/R− kidney transplant recipients, CMV-CMI (QuantiFERON-CMV) was detected in 6 patients who spontaneously resolved their infection without antiviral therapy [39]. These small-scale studies collectively suggest that assessment of CMV-CMI at the onset of low-grade asymptomatic CMV viremia may inform clinicians of the need to initiate, or withhold, preemptive antiviral treatment (Figure 1B) [38].

- *A robust CMV-CMI at the onset of low-level asymptomatic CMV reactivation suggests the potential for spontaneous viremia clearance that may not require preemptive antiviral drug treatment*.

### 3.5. Can I Use CMV-CMI to Guide the Duration of Antiviral Treatment of CMV Disease and Inform the Risk of Post-Treatment CMV Relapse?

**Figure 2:**
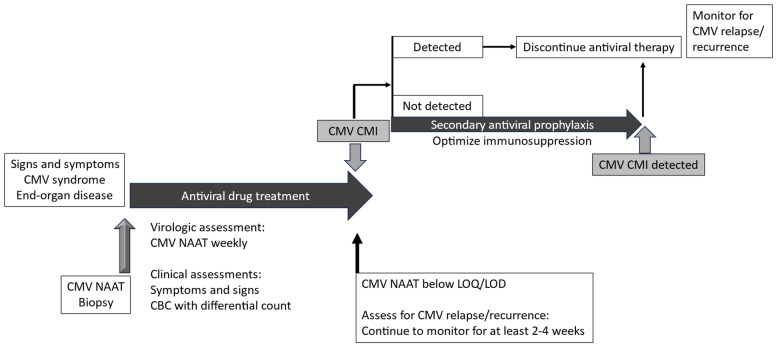
Treatment of CMV infection and disease that incorporates measures of CMV-specific cell-mediated immunity to complement viral load and clinical monitoring to guide the duration of antiviral treatment and the need for secondary antiviral prophylaxis. Footnote: CBC, complete blood count; CMV, cytomegalovirus; CMV-CMI, cytomegalovirus-specific cell-mediated immunity; LOD, limit of detection; LOQ, limit of quantification; NAAT, nucleic acid amplification test.

Intravenous ganciclovir or oral valganciclovir is the standard treatment for CMV infection and disease after SOT. The duration of antiviral treatment is highly individualized and guided by serial CMV NAAT (Figure 2). It is recommended that ganciclovir or valganciclovir treatment is continued until symptoms have resolved and the CMV viral load is undetectable in the blood for 1–2 weeks [4]. Despite this approach, recurrence of CMV infection occurs in about 20% of patients [20,40]. Anecdotal experience suggests that CMV relapse may be associated with an underlying severe degree of immunosuppression. This raises the question: can we use CMV-CMI as a complement to standard viral load testing to guide antiviral treatment of CMV disease?

In one study of 20 SOT patients treated with valganciclovir or IV ganciclovir for CMV infection or disease, none of the 18 patients who developed CMV-CMI (QuantiFERON-CMV) during treatment had relapse after stopping antiviral therapy [11]. In contrast, one of two patients who did not develop CMV-CMI had CMV recurrence [11]. Another study of 17 CMV-seropositive kidney transplant patients with CMV viremia demonstrated that patients with weak CMV-CMI (ELISPOT; <25 SFC/200,000 PBMC) were more likely to progress to CMV syndrome and require longer durations of antiviral treatment [41]. In an interventional study of 27 SOT patients receiving antiviral treatment for CMV infection (median viral load, 10,900 IU/mL), CMV-CMI (QuantiFERON-CMV) was measured at the time of viremia clearance [42]. Among the 14 patients (51.9%) with reactive CMV-CMI at viremia clearance, antiviral drug treatment was discontinued, and only 1 patient subsequently developed low-level asymptomatic viral recurrence. In contrast, 13 of 27 patients (48.1%) had no detectable CMV-CMI at viremia clearance, and they were provided secondary antiviral prophylaxis for 2 months. Despite secondary prophylaxis, CMV recurrence occurred in nine patients (69%), including one patient who developed UL97-mutant gancicloviR−resistant CMV [42]. This observation suggests that secondary antiviral prophylaxis should be complemented by immune optimization to prevent recurrent CMV infections (Figure 2). In another study of 39 patients who received preemptive antiviral treatment for mostly primary CMV infection, the only variable significantly associated with CMV recurrence after treatment was lack of CMV-CMI (intracellular cytokine staining and flow cytometry) [43]. Finally, among 44 CMV-seropositive heart recipients, viral relapse was associated with the failure to reconstitute CMV-specific immunity (QuantiFERON-CMV) after the resolution of the CMV infection [44].

- *CMV-CMI may be used to complement viral load testing to guide the duration of treatment of CMV disease. Ideally, antiviral treatment should be continued until the virus is undetectable and CMV-CMI is detectable*.

## 4. Limitations and Future Directions

The major hurdle to the adoption of CMV-CMI in clinical practice at this time is the lack of widespread availability of CMV-CMI assays. For example, QuantiFERON-CMV is not available for clinical use in the United States, although it is used in many centers in Canada, Europe, and Asia. Most CMV-CMI assays are available mainly in specialized referral centers, and many are still within research laboratories. Moreover, many CMV-CMI assays still lack clinical validation and standardization, which may be difficult to overcome due to the many differences in assays, processes, and platforms. It will be very difficult to standardize across all CMV-CMI assays, so there should be efforts to provide clinical validation for each of them. This means defining the optimal timing and frequency of testing for the various SOT risk groups and defining assay-specific thresholds of CMV immune competence.

Current CMV-CMI assays measure only CD4+ and/or CD8+ T cell function via interferon gamma release after ex vivo CMV antigenic stimulation. There are other aspects of pathogen-specific immune response that are not fully accounted for by these CMV-CMI assays. Other immune cells, including NK cells and cytokines other than interferon gamma, are not included in the currently available immune assays. It is possible that a more comprehensive measure of CMV-CMI that incorporates various aspects of the immune response may provide better insight into the CMV–host interactions and improve the clinical utility and interpretation of these assays. Finally, there is a need for more prospective, controlled, and interventional studies to support the promising role of CMV-CMI in CMV management in SOT recipients. Only a few prospective and interventional studies have been performed, although they are needed to support the suggested clinical applications of CMV-CMI, such as (1) pre-transplant prognostication of post-transplant CMV risk in CMV-seropositive transplant candidates, (2) post-transplant monitoring to guide the duration of antiviral prophylaxis in CMV-seropositive solid organ transplant recipients, (3) assessment of the need for preemptive antiviral treatment of asymptomatic CMV reactivation, (4) assessment of the duration of treatment of CMV disease, and (5) assessment of the risk of CMV relapse and the need for secondary antiviral prophylaxis.

## 5. Conclusions

This review highlights the role of CMV-CMI in the pathogenesis and outcomes of CMV infection after SOT. In general, a deficiency of CMV-CMI has been consistently correlated with an increased risk of post-transplant CMV infection, while highly reactive CMV-CMI is associated with protection from CMV disease. There are several CMV-CMI assays that are available for clinical use in different regions of the world, including commercial and laboratory-developed tests, but they are not yet standardized and have not been directly compared head-to-head in a controlled and comprehensive manner for the various clinical indications. Accordingly, there is no preferred CMV-CMI assay that can be widely recommended. Instead, one should consider using locally available, clinically validated CMV-CMI assays to predict the risk of post-transplant CMV and thus guide strategies for CMV disease prevention. Emerging data suggest that CMV-CMI can tailor the duration of prophylaxis among CMV-seropositive solid organ transplant recipients, predict the risk of relapse after treatment of CMV infection, and address the potential need for secondary antiviral prophylaxis. However, more interventional studies are needed to further validate the promising roles of CMV-CMI after SOT.

## Figures and Tables

**Table 1:** Clinical uses of Cytomegalovirus-Specific Cell-Mediated Immunity (CMV-CMI) assays in solid organ transplant recipients.

Clinical Scenario for Use of CMV-CMI Assays	Proposed Guidance	
Pre-transplant CMV-CMI to assess CMV risk post-transplant	CMV R−	CMV-CMI is not a useful tool. Almost all CMV-seronegative individuals have negative CMV-CMI, so pre-transplant testing has no prognostic role beyond what is provided by serology.
	CMV R+	CMV-CMI may be a useful tool for post-transplant CMV risk prediction in CMV R+ solid organ transplant candidates. A negative pre-transplant CMV-CMI portends a higher risk of post-transplant CMV infection. A positive pre-transplant CMV-CMI is associated with a lower risk of post-transplant CMV infection.
Post-transplant testing to assess CMV risk after transplantation, and guide CMV prevention strategy	CMV D+/R−	CMV-CMI can predict post-transplant risk in CMV D+/R− solid organ transplant recipients, but it is not cost-beneficial or practical when used during antiviral prophylaxis. Almost all CMV D+/R− solid organ transplant recipients have negative CMV-CMI during the period of antiviral prophylaxis. CMV-CMI may not be used to guide the duration of antiviral prophylaxis in CMV D+/R− solid organ transplant recipients.
	CMV R+	CMV-CMI may be useful for post-transplant CMV risk assessment in CMV R+ solid organ transplant recipients. A negative CMV-CMI is associated with a higher risk of CMV, while a positive CMV-CMI is associated with a lower risk of CMV infection. Serial CMV-CMI may be considered for individualizing the duration of CMV prophylaxis in CMV R+ solid organ transplant recipients. Antiviral prophylaxis may be stopped once a robust CMV-CMI is detected. CMV-CMI may be useful for determining the need for preemptive antiviral therapy of asymptomatic low-level CMV DNAemia. A robust CMV-CMI in a patient with asymptomatic low-level CMV DNAemia may lead to self-resolving infection.
Post-transplant guidance of duration of treatment for CMV infection and the need for secondary prophylaxis	CMV D+/R−	CMV-CMI may be a useful tool for post-transplant guidance of CMV treatment, as the development of robust CMV-CMI in CMV D+/R− transplant recipients implies effective immunity and suggests that treatment may be safely discontinued with a low risk of relapse or recurrence. Absence of CMV-CMI at end of treatment (viremia clearance) is correlated with CMV recurrence and suggests the need to consider secondary prophylaxis (and optimization of immunosuppression).
	CMV R+	CMV-CMI may be a useful tool for post-transplant guidance of CMV treatment, as the detection of robust CMV-CMI implies an effective immune reconstitution and safety in stopping antiviral treatment without the need for secondary antiviral prophylaxis. In selected CMV R+ solid organ transplant patients (e.g., highly immunosuppressed), CMV-CMI may be a useful tool for post-treatment guidance of the risk of CMV relapse (and the need for secondary prophylaxis).

Supportive evidence for this proposed guidance is discussed in the body of this article.
